# Recommendations on e-infrastructures for next-generation sequencing

**DOI:** 10.1186/s13742-016-0132-7

**Published:** 2016-06-07

**Authors:** Ola Spjuth, Erik Bongcam-Rudloff, Johan Dahlberg, Martin Dahlö, Aleksi Kallio, Luca Pireddu, Francesco Vezzi, Eija Korpelainen

**Affiliations:** Department of Pharmaceutical Biosciences and Science for Life Laboratory, Uppsala University, Uppsala, P.O. Box 591, SE-75124 Sweden; SLU-Global Bioinformatics Centre, Department of Animal Breeding and Genetics, Swedish University of Agricultural Sciences, Uppsala, Sweden; National Genomics Infrastructure, Science for Life Laboratory, Uppsala University, Stockholm, P.O. Box 1031, SE-17121 Sweden; Science for Life Laboratory, Uppsala University, Husargatan 3, Uppsala, SE-75123 Sweden; CSC - IT Center for Science Ltd., Espoo, P.O. Box 405, FI-02101 Finland; CRS4, Polaris, Loc. Piscina Manna Ed. 1, Pula, 09010 Italy; University of Cagliari, Cagliari, 09124 Italy; Science for Life Laboratory, Stockholm University, Stockholm, SE-17121 Sweden

**Keywords:** E-infrastructure, Next-generation sequencing, High-performance computing, Cloud computing

## Abstract

With ever-increasing amounts of data being produced by next-generation sequencing (NGS) experiments, the requirements placed on supporting e-infrastructures have grown. In this work, we provide recommendations based on the collective experiences from participants in the EU COST Action SeqAhead for the tasks of data preprocessing, upstream processing, data delivery, and downstream analysis, as well as long-term storage and archiving. We cover demands on computational and storage resources, networks, software stacks, automation of analysis, education, and also discuss emerging trends in the field. E-infrastructures for NGS require substantial effort to set up and maintain over time, and with sequencing technologies and best practices for data analysis evolving rapidly it is important to prioritize both processing capacity and e-infrastructure flexibility when making strategic decisions to support the data analysis demands of tomorrow. Due to increasingly demanding technical requirements we recommend that e-infrastructure development and maintenance be handled by a professional service unit, be it internal or external to the organization, and emphasis should be placed on collaboration between researchers and IT professionals.

**Electronic supplementary material:**

The online version of this article (doi:10.1186/s13742-016-0132-7) contains supplementary material, which is available to authorized users.

## Background

**Fig. 1:**
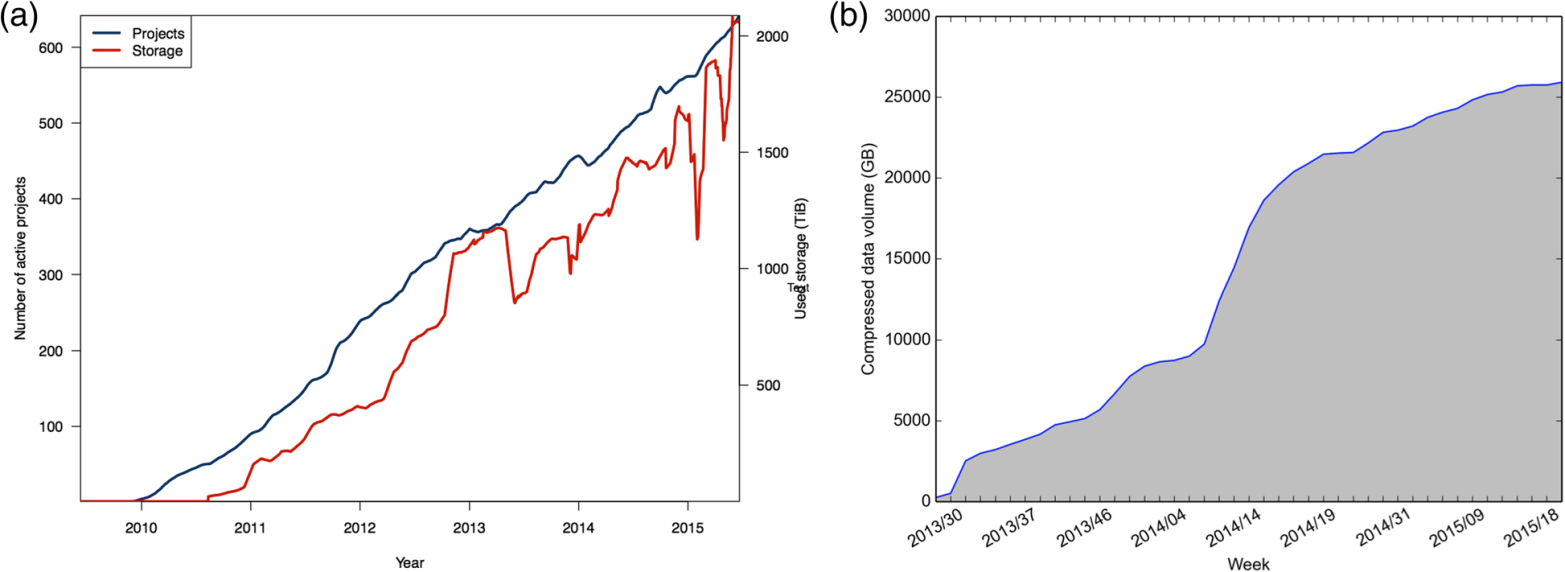
Active projects and used storage by bioinformatics projects. **a** UPPMAX HPC center in Sweden; **b** storage space dedicated to compressed sequencing data at CRS4. UPPMAX started logging storage utilization in 2011. We observe that the storage demand increases with the number of active projects. The irregularities in storage use are due to: at the end of 2012 a new storage system was installed, resulting in temporary data duplication as the systems were synchronized; at the beginning of 2015, the two sharp dips are due to problems with data collection. The storage usage plot from CRS4 has data ranging from mid-2013 to the first quarter of 2015. The plot only includes the space dedicated to storing compressed raw sequence data (fastq files; no raw data or aligned sequences), but still illustrates the upward trend in storage requirements

Massively parallel sequencing, also known as next-generation sequencing (NGS), has reduced the cost and increased the throughput of biological sequencing enabling the study of biological phenomena on a detailed level with great promise for improving clinical care [1–3]. Storing and analyzing the huge amounts of data generated by sequencing and other high-throughput technologies requires e-infrastructure providing high-performance computing and large-scale storage resources. Figure 1 and the work by Lampa et al. [4] illustrate the point, showing the growth in storage used for bioinformatics projects at UPPMAX in Sweden and at CRS4 in Italy. Note, however, that the best way to employ these resources in this context is open to debate [5]. In response to the phenomenal flood of next-generation sequencing data, the EU COST Action SeqAhead [6] was created with the primary objective of developing a coordinated action plan for the European life sciences community to deal with the data in an efficient and coherent manner using state-of-the-art bioinformatics.

This report summarizes the outcome of the discussions on e-infrastructures for NGS within the EU COST Action SeqAhead and provides general recommendations as well as a future outlook.

## E-infrastructure recommendations

**Fig. 2:**
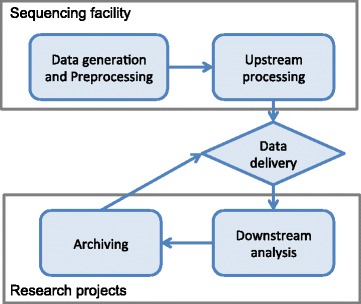
Overview of the different data analysis stages in a typical next-generation sequencing project with different requirements for e-infrastructures. Data is generated at the sequencing facility where it is preprocessed and commonly subjected to upstream processing that can be automated (such as alignment and variant calling). Data is then delivered to research projects for downstream analysis and archiving on project completion. Archived data can then be brought back as a new delivery when needed

Research projects using NGS have different e-infrastructure requirements for different stages in the data processing lifecycle. In this scenario, we can define two broad categories of actors: the data producers (e.g., sequencing core facilities), which aim to deliver high-quality data; and the research projects, which are focused on interpreting the data to solve biological problems. The lifecycle of the data may be considered to comprise five different stages with different e-infrastructure needs, as outlined in Fig. 2.

1. *Data generation and preprocessing.* Data is generated and subjected to initial preprocessing steps, such as conversion of raw data to standard formats (e.g., bcl2fastq conversion) and initial quality controls.
2. *Upstream processing.* The sequencing facility may perform a generic analysis that can be automated. This is also commonly called primary analysis (e.g., alignment, *de novo* assembly, etc.).
3. *Data delivery.* Data is transferred from the sequencing platform to the e-infrastructure of the research project.
4. *Downstream analysis.* Analysis is then performed that is specific to the research problem at hand. This is also called secondary analysis (e.g., trio/quad variant calling, gene annotation, etc.).
5. *Archiving.* The raw data and the data resulting from analysis are archived for a longer period.

We observe that the most common e-infrastructure components include high-performance computing (HPC) resources equipped with batch (queueing) systems, commonly connected to shared network-attached storage (NAS). Another e-infrastructure component that is gaining in popularity in NGS is cloud computing [7] on virtualized resources, and in this context we focus primarily on infrastructure as a service (IaaS). Three examples of e-infrastructures for NGS analysis in Sweden (UPPMAX), Finland (CSC) and Italy (CRS4) are available in Additional file 1. However, we note that there is a wide range of emerging commercial cloud services offering integrated platforms and software built on this technology. In the authors’ experience, grid computing has had little uptake in data-intensive bioinformatics.

### Data generation, preprocessing, and upstream processing

**Fig. 3:**
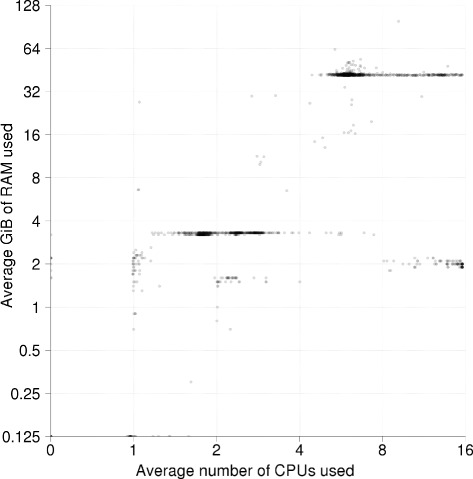
Average resource usage for the human whole genome sequencing pipeline at the National Genomics Infrastructure at SciLifeLab during the 6 month period May to October 2015. The pipeline consists of the GATK best practice variant calling workflow [33, 34] plus a number of quality control jobs. Each point in the figure is a job and the axes show the average number of CPUs and GiB RAM used by the corresponding job. The graph illustrates how this standard high-throughput production pipeline has a very clear resource usage pattern that does not achieve full CPU utilization on the 16 core nodes it runs on

The stages of data generation, preprocessing, and upstream processing are commonly carried out by sequencing and bioinformatics core facilities. Since the analysis in this phase is not specific to any given research project, it follows some general workflows that can normally be automated; there is a clear desire to automate them to ensure scalability and reproducibility. The extent of upstream processing also varies a lot between core facilities; for example, some do not provide much analysis, some only provide analysis for specific model organisms, whereas others have extensive analysis services available (see Fig. 3 for average resource usage for the human whole genome sequencing pipeline at the National Genomics Infrastructure, SciLifeLab, Sweden). The amount of data that must be handled at this stage is significant. For example, a single run of one Illumina HiSeq X sequencer outputs 16 whole human genomes with approximately 30 × coverage in 3 days, amounting to roughly 1.8 TB of data. Therefore an e-infrastructure connected to an X-Ten solution – i.e., ten HiSeq X sequencers – might be required to successfully process 36 TB of data per week, divided into two batches of 18 TB, for a total of 320 samples/week.

#### Recommendations

As the effort and costs to maintain a professional-grade e-infrastructure of compute and storage resources is considerable, our recommendation is that computational resources should be provided either using an HPC (batch) system or an IaaS approach that is maintained by a professional and dedicated unit. An alternative is to use a big data framework such as Hadoop with a distributed file system, which can work well on commodity hardware and also improve horizontal scaling. However, this approach requires specialized Hadoop-based software suites which, for NGS, are not as developed as for plain Linux systems [8]. In all cases where sensitive data is being processed, appropriate privacy measures need to be in place – especially when using external resources such as a public cloud provider [9].

**Fig. 4:**
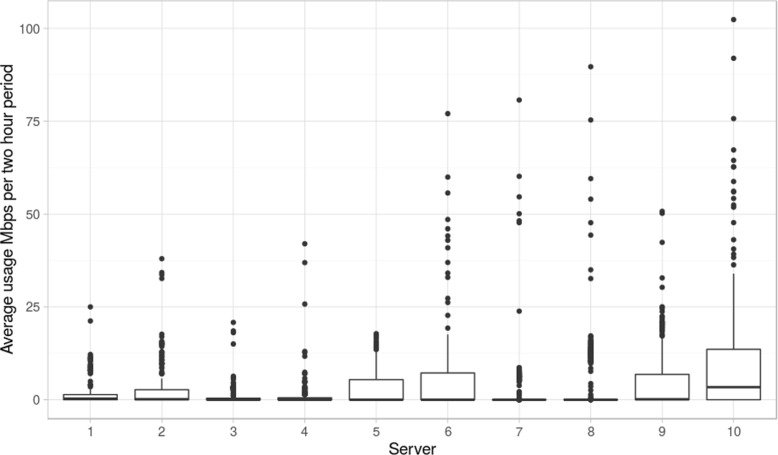
Average network usage for servers connected to sequencers. Average network usage (across a 2 hour window) measured during a 1 month period for ten servers with ten Illumina sequencers attached (one MiSeq, four HiSeq 2500, five HiSeqX) at the SNP&SEQ Technology platform. This data includes all traffic to and from the server, including writes from the sequencer and synchronization of data to other internal and external systems

There is a need for a networked storage system on the data producer side to which the instruments will write their data – for instance, see Illumina’s recommendations to set up an X-Ten or X-Five platform. It is important to have this storage system placed close to the sequencer(s) (in terms of network distance) to reduce the probability of data loss due to network outage. The average network usage of such a system will depend on the exact setup. We observe that for a setup with one server per instrument (see Fig. 4), the average network usage per server remains relatively low, only rarely exceeding 50 Mbps. This local storage solution buffers data during the instrument’s sequencing run. Due to the rate of data production, as soon as a run is completed the data should be quickly moved to an HPC or IaaS solution for further analysis to avoid filling the buffer storage during successive runs. To keep it safe, the raw data should also be backed up off site until it is delivered to clients.

Once processing begins, storage on the HPC side can easily become a performance bottleneck, and it is therefore important to equip the operation with a storage system that can provide high bandwidth and support many input/output (I/O) operations per second (IOPS). Furthermore, operations creating or removing a lot of files should preferably use local scratch disks on compute nodes rather than a shared file system due to high I/O load. The network needs to be able to sustain large data transfers to/from the preprocessing systems. At today’s price points, when buying new equipment we recommend investing in 10 Gbit Ethernet, which is quite fast without being tremendously expensive. Faster but more expensive interconnect between compute nodes (e.g., Infiniband) is more important in the cases where Message Passing Interface (MPI) programs are used (e.g., Abyss [10]), and these are not so common in upstream processing.

Regarding software, the specific software stack required depends on the particular needs of the operation and the users. Whatever the selection, it is important to record the parameters used in preprocessing as they can affect downstream analysis and results. In addition, we recommend using a workflow system for automating upstream processing [11].

### Data delivery

As a bare minimum, sequencing data is delivered from the sequencing facility to scientists. However, what is considered to be deliverable data from a sequencing run changes over time as technology and habits evolve. For instance, in earlier days, sequencing platforms stored and archived the actual images from Illumina sequencers. Nowadays, the image data is discarded after base calling which, at least with Illumina sequencers, happens right after the image has been acquired, leaving on disk only the bases and intensities that were sequenced. Currently, sequencing facilities at a minimum typically deliver fastq files with sequences and base qualities – the direct result of the base calling procedure. Facilities can go further and perform preprocessing and upstream processing to deliver aligned data (commonly BAM files) and variant data (commonly VCF files) to the customers. These supplementary results need to be delivered to users, often shortly after the basic data is delivered. Therefore, a data delivery system needs to be in place in order to track the status of projects and deliveries.

#### Recommendations

We recommend delivering sequences and base qualities to customers as standard compressed fastq files, plus a quality control report and results from at least basic processing (e.g., alignment), since this process can be automated. Results should preferably be delivered along with all the provenance information needed to reproduce them, i.e., the exact computational steps and parameters used. There is currently no standardized format for data provenance in NGS, but effective solutions include exporting a Galaxy [12] history or a Chipster [13] session, since these record the sequence of programs used, along with the software version and parameters. As a minimum, the provenance data should report the versions of software, databases, and references, and any workflows and workflow engines that were used.

The requirements of the data delivery stage depend on the specific situation at hand, and whether upstream and downstream processing are taking place on the same e-infrastructure. For organizations that perform their own sequencing and use a single e-infrastructure, ‘delivering’ simply means making the directory containing the data accessible to downstream users. However, the operation can be more challenging when the two phases run on different infrastructures. This case is likely most common when the two phases run at different centers, but it can also happen when all users are under the same roof, since it can be advantageous to use separate e-infrastructures for upstream processing and downstream analysis as these operations have different usage patterns and hence require different system configurations (e.g., memory size, storage bandwidth, etc.). To serve users on separate computing resources within a single organization, the e-infrastructure should include a high-bandwidth network to allow for data delivery. To deliver to users outside the organization, if possible the centers should try to equip themselves with a high-bandwidth internet connection and use specialized file transfer tools, such as Aspera [14] and GRIDftp [15]. Alternatively, for partners invested in a long-term collaboration, the upstream organization can consider providing the downstream users access to its computational resources near the data storage – a solution adopted by the European Molecular Biology Laboratory European Bioinformatics Institute (EMBL-EBI) Embassy cloud [16]; in this manner, the most voluminous data never needs to be transferred from where it was generated. Finally, the old but reliable method of delivering data on physical disk should not be discounted; what it lacks in practicality it makes up for with excellent bandwidth.

### Downstream analysis

Because of the diversity in downstream analysis pipelines, trying to support them all on a particular e-infrastructure is challenging. This part of the analysis typically requires a combination of factors that is difficult to achieve: high flexibility and reproducibility, as well as significant computing and storage capacity. There have been many attempts to improve the situation (e.g., software suites, web services, workflow systems, Linux distributions tailored for bioinformatics). In the authors’ experience, none of these proposed solutions have ever managed to garner a critical mass of adopters, and in fact they are rarely used by core facilities. The authors are a little puzzled as to why this is the case, but one reason could be the lack of agreed-upon standards and the multitude of solutions available [17]. Moreover, especially when working with complex organisms, simple workstations are insufficiently powerful to perform the desired analyses so, whatever the software solution used, it needs to run on high-performance e-infrastructures. In part, the significant computational requirements are due to the fact that current bioinformatics tools are often inefficient because their development is often driven by the urgent need to find solutions to biological problems; this leaves little time for more sophisticated implementations which, though more efficient, would require a much more significant investment in development. Further, HPC has traditionally been driven by physics, while biology is a newcomer to the field. This has resulted in traditional HPC infrastructure configurations that are sometimes an ill fit for bioinformatics workloads. Generally, the computations involved in bioinformatics are more high-throughput than high-performance; e.g., the same type of computation is applied to a large number of samples rather than one long-running simulation experiment. This approach tends to be more data intensive instead of computationally intensive like most physics analyses.

#### Recommendations

We recommend that computational resources should use HPC systems with batch support or cloud resources (IaaS) – the latter are useful to provide an elastic infrastructure to users that have bursty workloads, making a large number of nodes available only for the time they are needed. Bioinformatics tasks typically require a lot of cluster-attached storage and random-access memory (RAM). Assembly is a prime example of a bioinformatics task that requires machines with lots of memory, sometimes up to several terabytes of RAM [18].

Web services and web-based applications are a good way to organize and provide high-level functionality to users. Example uses of this technology are applications to provide users with access to administrative operations (for instance, to install new tools), giving system administrators the freedom to run many different computational frameworks (slurm, mesos, Hadoop, etc.) in parallel, and running high-level analysis workflow applications such as Chipster and Galaxy. Incidentally, we observe that frameworks such as Hadoop are not much used in downstream analysis.

As storage requirements are high in NGS, we recommend fast cluster-attached storage for downstream analysis. It is important to back up important files, but due to project size it might be (economically) unfeasible to back everything up. Temporary files can make NGS projects grow five to ten times on disk, so when deciding what to keep, one should weigh the time/cost to recompute intermediate results versus the cost to store them and the probability that they might be needed again. Our recommendation is to keep only the raw compressed fastq or bcl files, along with the necessary metadata describing the samples, for long time storage. This data is not only necessary to fully replicate the experiment, but it is often a mandatory requirement in case of quality assured facilities. Depending on the application, it might also be possible to commit the results of primary and/or secondary analysis (e.g., expression levels, variants, etc.) to long-term archives. On the other hand, all intermediate files (e.g., alignment) need to be removed as quickly as possible, but all the provenance information needed to reproduce them needs to be stored and associated with the raw data.

If providing a general e-infrastructure to scientists, a wide range of bioinformatics tools needs to be installed in order to cater to the various requirements of different bioinformaticians. Virtual machine images can simplify provisioning of environments, avoiding cumbersome installation of tools with complex dependencies. On shared systems with many users, managing software installations and upgrades might require dedicated personnel.

As previously mentioned, current downstream analysis often makes inefficient use of e-infrastructure resources. Educating users to improve the efficiency of their operations should help improve the situation, as should investment in tools that use computational resources more efficiently. We see the possibility of using workflow systems to improve resource utilization. In many cases such solutions (e.g., Galaxy and Chipster) can excel over simple in-house scripts when it comes to decomposing analysis into smaller parts that can be run separately with adequate resource allocations [17]. Also, cloud systems allow for overcommitting virtual CPUs (sharing of compute cores) which could improve the situation for some types of inefficient bioinformatics jobs, but due to their variable memory usage patterns we do not think this will work as well as it has for general IT server workloads (e.g., web servers). On the other hand, big data frameworks, such as Hadoop and especially Spark, would allow efficient resource usage in cloud systems.

### Long-term storage and archiving

After downstream analysis, raw data, results, and temporary files often need to be moved from cluster-attached storage to medium- or long-term storage. Medium-term archival can be required while waiting for publication, or because of a pause in the project (for instance, while waiting for data from additional samples to become available). Upon project completion, data typically goes into long-term archival and datasets may need to be published online.

#### Recommendations

For long-term storage and archiving the focus is on storage reliability, not performance. Disk- and tape-based solutions are currently the cheapest solution for this task. We suggest the use of specialized compression for long-term storage, despite the fact that many common bioinformatics tools do not read these formats directly [19]. Various compression tools exist [20, 21]; of these, CRAM [22] may be the most popular thanks to the support from EMBL-EBI. When archiving upon project completion, we recommend only keeping the raw data and final results, not the intermediate data. However, it is important to store the complete analysis workflow with all parameters and software versions to ensure reproducibility of the work and the ability to regenerate the deleted files, if needed. Also, to help devise rational archival policy one should estimate the *total cost* of long-term data storage and compare it with the cost of regenerating the data – even considering resequencing if it is possible to store or obtain new samples.

## Discussion and outlook

The most common e-infrastructure for NGS data management and analysis is currently an HPC cluster with a network-attached storage system, running the Linux operating system, and with bioinformatics tools installed. However, we observe a trend that IaaS solutions are becoming available and have seen some adoption in NGS analysis. We are pleased to observe that e-infrastructures are increasingly planned and procured along with the data-generating instruments (such as sequencers) that they support – unlike just a few years ago, when the data management infrastructure was in many cases ignored and not included in grant applications. Nowadays, the costs for e-infrastructure are visible and form a big part of NGS investments. However, the time to plan, procure, install, and test e-infrastructure is considerably longer than the time necessary to obtain an operational data-generating instrument, and it is not uncommon that new sequencers are acquired before the supporting e-infrastructure is fully deployed, forcing them to run at reduced capacity.

Once the e-infrastructure is deployed, we find that its typical users have limited experience with HPC environments and large-scale file systems. They find queueing systems to be an obstacle and also do not often perceive the costs of production-grade hardware and its maintenance. A lack of information or training is likely partly to blame for these problems. For the same reason, biology-oriented users of HPC systems for NGS analysis often have very high expectations on storing data, in the sense that it is not uncommon for them to expect to store both raw data and derived files for a long time. While it may be desirable to reprocess samples in the future as new, improved analysis techniques appear (e.g., aligners and assemblers), this can still be done by restarting the processing from the raw data, albeit with longer computing times. Thus, it is important that users consider the trade-off between storage space and recomputing time, as well as considering data reduction as other scientific disciplines have done (for instance, physics and astronomy) [23, 24]. We recommend that users undergo some basic training to help improve their usage patterns. Furthermore, users should pay, at least in part, for services based on their actual usage, establishing a direct link between usage and cost. This strategy serves especially to raise user awareness about infrastructure costs; in our experience, systems without user fees lead to users not being diligent and responsible with their allocations of computing and storage resources. In the users’ defense, apart from the frequently seen inefficient use of storage space, they are sometimes not directly responsible for inefficient use of computing resources. In fact, few bioinformatics tools are made with HPC architecture in mind. For instance, support for MPI is rare; instead, most programs are written to run on a single node, reading and writing data to locally *accessible* files (usually not stored locally on the machine). Given that common HPC architecture does not offer good data locality, since the data usually needs to travel over the network to be processed, this operational pattern can be overly taxing on the cluster network and NAS. To make matters worse, in many cases these tools do not use memory and CPU resources efficiently. In large-scale operations, these issues become particularly relevant as the number of concurrent instances of these programs is multiplied.

The common temporary solution for the efficiency problem is to buy and install more powerful e-infrastructure. In the long term, we see two better solutions to achieve scalability. 1) Optimize bioinformatics tools for HPC or IaaS resources. We believe that one crucial step in this direction is the emergence of commercial actors such as Curoverse [25] and Seven Bridges Genomics [26]. 2) Port bioinformatics tools to massively parallel computing (big data) frameworks such as Hadoop MapReduce, Spark, and Flink, with distributed file systems such as the Hadoop file system (HDFS). Big data frameworks have been shown to be successful for some bioinformatics problems, and we believe they hold a promising future for large-scale bioinformatics applications. Work is being done in this direction, with projects such as ADAM [27], Seal [28], and Hadoop-BAM [29] providing partial bioinformatics solutions that exploit big data frameworks. Notwithstanding these efforts, Hadoop and similar frameworks are not compatible with conventional HPC and storage resources so they require considerable effort to adopt. Furthermore, software needs to be specifically written to run on those platforms and, at least at the moment, the software available does not yet span the operations required by typical NGS pipelines. We believe that, in their current form, these solutions are not silver bullets for NGS analysis, but they should be further developed as they hold a lot of promise.

These big data approaches, and in particular distributed file systems, offer significant advantages to the data-parallel and data-intensive computing found in NGS because they offer much better data locality than the HPC cluster architecture. For this reason, we think that this technology has a place within the e-infrastructure for NGS, though the lack of compatible bioinformatics tools represents a hurdle that needs to be overcome for any significant level of adoption to occur. This development front is not very active, however, presumably because on average bioinformatics software is under continuous development, while this type of compatibility work is more suitable to stabilized software packages.

The rapidly evolving software ecosystem in NGS also makes the management of software installations a challenge for e-infrastructure providers. Keeping up with new installation requests and frequent software updates requires a significant amount of work. Virtual machines can help simplify the provision of the latest tools to users, while keeping an archive of older machine images can be used as part of a set of measures to ensure the reproducibility of past results. We promote the sharing of virtual machine images using catalogs such as BioImg.org [30]. Another recent relevant technological development is container technology, such as Docker [31], which can be used to package analysis tools and data to ensure easy deployment and reproducibility of the analysis. While this technology is still to be widely adopted, it holds promise for the future.

The topic of data-intensive e-infrastructures relevant to NGS analysis is an active area of development, and while HPC is currently the most common e-infrastructure for NGS, cloud-based systems are becoming increasingly common. Recent developments also support cloud-based HPC where a system such as OpenStack can provision HPC clusters, even on bare metal (i.e., without a host operating system [32]). This strategy combines the power and flexibility of virtualization with the performance of HPC and looks to be a very promising path for future e-infrastructures for NGS analysis.

## Conclusions

With the rapid development of NGS technology over the past few years, NGS data analysis has been evolving quickly to keep pace with a constant stream of new and updated software. We have not reached a plateau yet but things are slowing down, such as in the case of alignment tools. From an e-infrastructure perspective, it is important to understand the effort and costs involved in supporting large-scale NGS data analysis, and that setting up a self-built e-infrastructure that can be sustained over time is challenging and also questionable from an economic perspective. Significant attention needs to be paid to educating users, and it is also important to make infrastructure costs visible to bioinformaticians and principal investigators. International efforts are needed to standardize what software to use and how to automate processes, and to develop best practices that are accepted by the community. It is also important to strengthen the connection between biologists, bioinformaticians, computer scientists, and system administrators to enable more rigorously designed, tested, and deployed software programs that make better use of computational resources.

### Summary of recommendations

- It is important to have detailed plans for the e-infrastructure when investing in NGS, and to keep in mind that procuring computational hardware can take more time than procuring NGS equipment.
- Computational resources for NGS analysis should be provided by a professional service unit either as high-performance computing or infrastructure as a service.
- Data should be in compressed formats at all times, and monitoring tools should preferably be in place for this.
- Support and training are key components in addition to the e-infrastructure and should not be underestimated.
- Shared file systems can easily become a bottleneck in analysis; it is important to provide high I/O bandwidth and operations per second as well as scratch disks on compute nodes to be able to sustain a large number of concurrent NGS analyses.
- Workflow systems are recommended for upstream processing to ensure structured description of primary analysis.
- Implement user fees or make costs visible to end users.
- Big data frameworks and distributed file systems are promising technologies but are not currently compatible with most bioinformatics tools and need further development before mainstream adoption.

## Additional file

Additional file 1 Description of e-infrastructures at UPPMAX, CSC, and CRS4. (PDF 91 kb)
