# Supplementary material for: Recommendations on e-infrastructures for next-generation sequencing
Source: Gigascience. 2016 Jun 7;5:26. doi: 10.1186/s13742-016-0132-7 (PMC4897895; doi:10.1186/s13742-016-0132-7)
Supplement: Additional file 1 — Description of e-infrastructures at UPPMAX, CSC, and CRS4. (PDF 91 kb) [file 13742_2016_132_MOESM1_ESM.pdf]

Description of e-infrastructures

August 2, 2015

UPPMAX

UPPMAX - Uppsala Multidisciplinary Center for Advanced Computational Science, is one of six supercomputer centres in Sweden. Compared to the others it is a medium sized center that currently has 4 cluster computer systems.

The biggest cluster is a HP cluster with 208 nodes mainly used for bioinformatics. The standard node has 16 cores (dual Intel Xeon E5-2660), 128GiB RAM, and 4TiB local storage. A portion of the nodes are high-memory nodes, 17 nodes with 256GiB RAM and 17 nodes with 512GiB RAM.

The second biggest cluster is aimed at general HPC users, consisting of 160 nodes with 16 cores (dual AMD Opteron 6220), 64GiB RAM, and 2TiB local storage. There are 4 nodes equipped with nVidia Tesla S2050 cards.

The other two systems are 1) a high-memory node with 64 cores and 2TiB RAM, and 2) an old cluster (350 nodes, 8 cores per node, 24GiB RAM) that has been repurposed as a cloud development system to start testing the possibilities of OpenStack.

UPPMAX is not involved in any of the DNA sequencing, but serves as the default delivery method for the large part of all sequencing data being produced in Sweden. The project within UPPMAX that handles that part is called UPPNEX - UPPMAX Next Generation Sequencing and Storage [1]. The actual sequencing is performed by a Swedish government research project called SciLifeLab - Science for Life Laboratory (`http://www.scilifelab.se`).

UPPMAX employs several application experts in various fields, bioinformatics among them, that are active researchers in their fields, that can help users with both practical and conceptual problems. This has been a key point in helping users with their analyses.

CSC

CSC - IT Center for Science is a national computing centre that provides IT support and resources for universities, research institutes and companies. CSC does not run sequencing operations, but provides IT infrastructure at various levels to support NGS data production and processing at various institutions in Finland.

CSC and ELIXIR Finland have created a dedicated cloud environment for Finnish Institute for Molecular Medicine (FIMM). Through cloud and network virtualisation the local cluster at FIMM can be seamlessly extended with capacity from CSC data center. This allows to easily scale data processing capacity according to growing sequencing needs. CSC also provides public cloud computing environment called cPouta and is developing ePouta cloud environment for sensitive data. Cloud environments are based on OpenStack and allow allocation of resources dynamically on demand (Infrastructure-as-a-Service).

As a national bioinformatics facility CSC serves a large number of users, the majority of whom have bio/medical background and no programming skills. CSC develops a growing portfolio of higher level cloud services that allow downstream analysis of sequencing data. We enable users to work independently by providing training and user friendly interfaces. An example is the Chipster software, developed at CSC and offered with a Software-as-a-Service model. Chipster provides a graphical user interface to a large suite of analysis tools for NGS data [2].

CRS4

CRS4 is a government research center that hosts a high-throughput sequencing facility with three Illumina HiSeq2000, making it the largest NGS platform in Italy. It has participated in large-scale population-wide genetic studies [3, 4] and provides sequencing services for external collaborators and clients. All the data produced by the sequencing laboratory undergoes some degree of processing in the computing center, spanning from quality control and packaging to reference mapping and variant calling – as required for the specific project.

To handle the sequence platform's data production capacity, in addition to significant software development efforts [5, 6, 7, 8], the center had to be equipped with adequately infrastructure. The sequencing machines have a direct network link with the computing center (in an adjacent building)

which houses all storage and computing resources.

The main computing resource is a 384-node HPC cluster, which is accessed through the Open Grid Engine batch queuing system. The nodes are not well-equipped by today's standards (each node has only 16 GB RAM, 8 cores, 250 GB of local disk space), but thanks to their high number and the scalability of the software system the pipeline's processing throughput is much higher than the data production capacity. The nodes are networked through 1 GbE, with two 40-Gbit core routers. The storage infrastructure is constituted by a single shared parallel file system, which is accessible by all cluster nodes and also by the sequencing machines. The storage system provides a usable capacity of 4 PB and runs the GPFS.

References

[1] Lampa, S., Dahlö, M., Olason, P.I., Hagberg, J., Spjuth, O.: Lessons learned from implementing a national infrastructure in sweden for storage and analysis of next-generation sequencing data. Gigascience **2**(1), 9 (2013). doi:10.1186/2047-217X-2-9

[2] Kallio, A., Tuimala, J., Hupponen, T., Klemelä, P., Gentile, M., Scheinin, I., Koski, M., Kaki, J., Korpelainen, E.: Chipster: user-friendly analysis software for microarray and other high-throughput data. BMC Genomics **12**(1), 507 (2011). doi:10.1186/1471-2164-12-507

[3] Orrù, V., Steri, M., Sole, G., Sidore, C., Virdis, F., Dei, M., Lai, S., Zoledziewska, M., Busonero, F., Mulas, A., Floris, M., Mentzen, W.I., Urru, S.A.M., Olla, S., Marongiu, M., Piras, M.G., Lobina, M., Maschio, A., Pitzalis, M., Urru, M.F., Marcelli, M., Cusano, R., Deidda, F., Serra, V., Oppo, M., Pilu, R., Reinier, F., Berutti, R., Pireddu, L., Zara, I., Porcu, E., Kwong, A., Brennan, C., Tarrier, B., Lyons, R., Kang, H.M., Uzzau, S., Atzeni, R., Valentini, M., Firinu, D., Leoni, L., Rotta, G., Naitza, S., Angius, A., Congia, M., Whalen, M.B., Jones, C.M., Schlessinger, D., Abecasis, G.R., Fiorillo, E., Sanna, S., Cucca, F.: Genetic variants regulating immune cell levels in health and disease. Cell **155**(1), 242–256 (2013). doi:10.1016/j.cell.2013.08.041

[4] Francalacci, P., Morelli, L., Angius, A., Berutti, R., Reinier, F., Atzeni, R., Pilu, R., Busonero, F., Maschio, A., Zara, I., Sanna, D., Useli, A., Urru, M.F., Marcelli, M., Cusano, R., Oppo, M., Zoledziewska, M., Pitzalis, M., Deidda, F., Porcu, E., Poddie, F., Kang, H.M., Lyons,

R., Tarrier, B., Gresham, J.B., Li, B., Tofanelli, S., Alonso, S., Dei, M., Lai, S., Mulas, A., Whalen, M.B., Uzzau, S., Jones, C., Schlessinger, D., Abecasis, G.R., Sanna, S., Sidore, C., Cucca, F.: Low-pass DNA sequencing of 1200 Sardinians reconstructs European Y-chromosome phylogeny. Science (New York, N.Y.) **341**(6145), 565–569 (2013). doi:10.1126/science.1237947

[5] Pireddu, L., Leo, S., Soranzo, N., Zanetti, G.: A hadoop-galaxy adapter for user-friendly and scalable data-intensive bioinformatics in galaxy. In: Proceedings of the 5th ACM Conference on Bioinformatics, Computational Biology, and Health Informatics. BCB '14, pp. 184–191. ACM, New York, NY, USA (2014). doi:10.1145/2649387.2649429. http://doi.acm.org/10.1145/2649387.2649429

[6] Cuccuru, G., Leo, S., Lianas, L., Muggiri, M., Pinna, A., Pireddu, L., Uva, P., Angius, A., Fotia, G., Zanetti, G.: An automated infrastructure to support high-throughput bioinformatics. In: High Performance Computing Simulation (HPCS), 2014 International Conference On, pp. 600–607 (2014). doi:10.1109/HPCSim.2014.6903742

[7] Pireddu, L., Leo, S., Zanetti, G.: Seal: a distributed short read mapping and duplicate removal tool. Bioinformatics **27**(15), 2159–2160 (2011). doi:10.1093/bioinformatics/btr325. http://bioinformatics.oxfordjournals.org/content/27/15/2159.full.pdf+html

[8] Leo, S., Zanetti, G.: Pydoop: A python mapreduce and hdfs api for hadoop. In: Proceedings of the 19th ACM International Symposium on High Performance Distributed Computing. HPDC '10, pp. 819–825. ACM, New York, NY, USA (2010). doi:10.1145/1851476.1851594. http://doi.acm.org/10.1145/1851476.1851594
